# Targeted and Personalized Therapy for Difficult Benign Brain Tumors: A Review

**DOI:** 10.3390/jpm16030170

**Published:** 2026-03-21

**Authors:** Polina Chliapnikov, Mark Bernstein

**Affiliations:** Division of Neurosurgery, Toronto Western Hospital, University Health Network, Toronto, ON M5T 2S8, Canada; mark.bernstein@uhn.ca

**Keywords:** biomarkers, methylation, molecular profiling, personalization, targeted therapy

## Abstract

Background: Difficult benign intracranial tumors (including meningiomas, schwannomas, neurofibromatosis-related tumors, and pituitary neuroendocrine tumors) have substantial morbidity in patients. Due to their limited treatment options, there is a need for individualized treatment beyond histological and surgical approaches. Objective: To summarize how novel treatment innovations have been implemented for these tumors, meningiomas and schwannomas are prioritized, followed by NF-associated neoplasms, and then pituitary neuroendocrine tumors in comparison to low-grade gliomas. Methods: We summarize the current knowledge relating to targeted therapies for gliomas, meningiomas, schwannomas, neurofibromatosis (NF) tumors, and pituitary neuroendocrine tumors to investigate an individual’s treatment options for difficult benign brain tumors. This review synthesizes evidence on tumor genomics and molecular markers, supported by methylation-based classification, immunohistochemistry, and functional assays, emphasizing current clinical applications. Evidence Synthesis: The recent data show that DNA methylation-based models can predict post-surgical outcomes and radiotherapy responses, enabling risk stratification and radiotherapy benefit prediction. Early signals support target-directed treatment, including cMET blockade that radiosensitizes NF2 schwannoma models, brigatinib-associated tumor shrinkage in NF2-deficient models, and PitNET organoid data. Conclusions: We support clinical decision-making that utilizes molecular profiling with functional testing to guide targeted treatment. We also identify evidence gaps such as biomarker-defined prospective trials that are needed for broader clinical implementation.

## 1. Introduction

Intracranial tumors that are histologically benign or low grade can still be difficult due to their location relative to major nerves and vessels as well as their occasionally aggressive behavior. Tumors are considered difficult when they cannot be safely or completely resected, are repeatedly recurring, accumulate treatment comorbidities, or present with molecular features associated with higher recurrence risk. Such tumors (notably gliomas, meningiomas, schwannomas, neurofibromatosis (NF)-related tumors, and pituitary neuroendocrine tumors (PitNETs)) may arise in eloquent cranial areas. They represent a substantial proportion of central nervous system (CNS) tumors, as non-malignant tumors comprise ~73.7% of CNS tumors according to the most recent Central Brain Tumor Registry of the United States (CBTRUS) report [1]. Seizures, progressive neurological deficits, visual compromise, hearing loss, endocrine dysfunction, cranial neuropathies, and several other morbidities can evolve from tumor growth, making personalized treatments key for improving survival and quality of life. The CBTRUS reports that 91.7% of patients have a 5 year survival rate for non-malignant tumors, supporting high prevalence and survivorship needs. Within the same CBTRUS dataset, meningiomas are recorded to be the most common benign histopathology (42.6% of all tumors; 54.7% of non-malignant tumors), while gliomas account for 22.2% of all tumors [1]. PitNETs (formerly known as pituitary adenomas) have a prevalence of about 76–116 per 100,000 people [2], while NF-related tumors and schwannomas have a frequency of about 1 in 3164 [3] and about 4.4 per 100,000 people [4], respectively. In this review, low-grade gliomas (although these are not truly benign tumors) are included as comparators, as their molecular taxonomy and biomarkers have more mature therapeutic translations.

Precision medicine refers to tailoring clinical decisions—including diagnosis, prognosis, surveillance, and therapy selection—to specific patient biology, rather than by relying on tumor population averages or morphology. In neuro-oncology, this approach is operationalized through molecular, epigenetic, and imaging biomarkers that stratify patients into groups with significantly different risks or expected treatment benefits. In the context of this paper, these markers include DNA methylation classes, copy-number alterations (risk biology), and immunohistochemistry protein markers. Together, such identifiers shift treatment decisions away from tumor class and toward biological stratification. There are three main levels of personalized tumor therapy: molecular taxonomy (genomics and mutations), biomarkers (proteins, imaging, immune microenvironment compositions), and functional precision oncology (patient-specific ex vivo tumor testing). For most difficult benign intracranial tumors, primary management typically consists of maximal safe surgical resection, with radiotherapy considered according to the residual disease, recurrence risk, grade, and clinical context. In current practice, emerging biomarker-informed and individualized therapies most often have an adjunctive role, particularly in residual or recurrent tumors, in higher-risk meningiomas such as WHO grades 2 and 3 disease, and in syndromic conditions such as NF1 and NF2 where patients may develop multiple tumors over time [5]; however, histology and conventional grading alone cannot reliably assess the heterogeneity associated with difficult benign tumors. In 2021, the fifth edition of the World Health Organization (WHO) classification of tumors of the CNS expanded the role of molecular parameters in the diagnosis and grading of such tumors [6], since genomic DNA methylation profiling has emerged as a fundamental method for integrated tumor diagnosis, changing about 12% of CNS tumor identification and having direct clinical impact [7]. Molecular profiling in difficult benign intracranial tumors aims to promote sharper diagnosis, more accurate risk stratification, and exposure of drug treatment pathways. As such, personalized therapies become plausible when treatments are identified based on the tumor-type biomarkers (through molecular profiling). Such clinically useful marker systems include driver alterations, copy-number patterns, immunohistochemistry, epigenetic classifiers (notably DNA methylation), radio genomic signatures, and functional assays of specific tumors [8].

By focusing on clinically actionable innovations—specifically methylation-based classification, risk models, tumor genomics, biomarkers, radiogenomic signals, immunohistochemistry, and functional chemosensitivity—tumor treatments can be personalized on a genetic marker level [8]. This review asks the following research question: how can integrated molecular profiling and functional testing be used to guide individualized, clinically implementable treatments for difficult benign intracranial tumors beyond surgery? As such, we aim to synthesize the current evidence on how molecular profiling and functional testing methods have been impactful in personalizing treatment for difficult benign intracranial tumors.

## 2. Materials and Methods

This article was designed as a structured narrative review, focusing on difficult benign CNS tumors—primarily meningiomas, schwannomas, NF-related tumors, and PitNETs—with low-grade gliomas serving as a comparator due to their more mature translations. The objective was to provide a summary of the most prominent current treatments in difficult benign CNS tumor treatment and the biomarkers used to develop them.

Search Strategy, Eligibility, and Evidence Synthesis:

We focused on prospective clinical trials, randomized trials, translational studies with mechanistic validation (including xenografts, genetically engineered models, organoids, and ex vivo systems), and large-scale molecular modeling studies with clinical endpoints. The literature base included targeted database searches in PubMed/MEDLINE, EMBASE, and Scopus to collect peer-reviewed articles on tumor biomarkers and chemosensitivity testing. All searches were limited to English-language sources, and the literature search was concluded on 2 February 2026.

Eligibility was defined by both the tumor type (gliomas, meningiomas, schwannomas, NF-related tumors, and PitNETs) and study quality. The study quality was determined by the presence of clinically interpretable endpoints, including progression-free survival (PFS), overall survival (OS), response rates, disease stability, toxicity, and functional endpoints. Preclinical and molecular classifying studies were also included when they provided mechanistic validation or modeling for any existing therapies or linked molecular frameworks.

From the included studies, data on tumor content, cohorts, markers, therapeutic interventions, clinical endpoints, and assay modality were extracted. The evidence synthesis followed a template that recorded the marker class (drivers, receptors, mutations, methylation-defined risk groups, signaling, and phenotype), medical decision modification, developed treatment, and clinical impact.

Study Actionability:

Due to the exploratory nature of such studies, few have targeted therapies entering stage III testing, and fewer have been translated into readily available therapies [9]. Actionability was dependent on the progression of the study and the impact the finding had on current tumor treatments. High actionability would be biomarker-defined trials in which the marker directly impacts therapy selection, moderate/emerging actionability would be preclinical phase II implementations which still require prospective validation to link assays with patient outcomes, and low/negative actionability would be early targeted therapies that showed limited, no, or negative tumor response to treatment.

## 3. Results

DNA methylation is a prevalent model for post-surgical outcomes and radiotherapeutic responses in the treatment of difficult benign brain tumors and is the most mature for meningioma treatment. A multicenter analysis by Sahm et al. showed that DNA methylation-based tumor classes predicted tumor recurrence and prognosis more powerfully than the WHO grade, supporting the need for methylation-informed stratification of CNS tumors, specifically meningiomas [10]. Subsequent work by Nassiri et al. validated methylation-based nomograms for recurrence prediction factors in large cohorts [11]. DNA methylation can help resolve diagnostically equivocal cases and stratify recurrence chance beyond conventional grading techniques [7].

Gliomas:

This paper focuses on benign tumors, but a brief discussion of gliomas can help inform and contextualize this topic. The WHO 2021 classification reframes adult-type diffuse gliomas as biologically defined tumors using core molecular features—specifically isocitrate dehydrogenase (IDH) mutation status and 1p/19q codeletion—rather than relying on morphology alone. WHO 2021 also embedded molecular criteria into grading, such that alterations (including deletions and amplifications on gene loci) can determine or upgrade a tumor grade to more accurately reflect the necessary treatment (Table 1) [12]. Also, epigenetic profiling adds a complementary classification that can clarify diagnostically equivocal cases and refine risk groups between similarly appearing histologies—allowing for reproducible prognostics that are not subjective to the visual prognosis of individual doctors [7].

A study by Hegi et al. showed that the treatment of grade 4 gliomas (glioblastomas) with O6-methylguanine-DNA methyltransferase (MGMT) promoter methylation is a clinically actionable predictive biomarker. Patients whose tumors have methylated MGMT promoters derive greater benefit from adding temozolomide (an oral chemotherapeutic agent) to standard radiotherapies than patients with unmethylated MGMT [13]. Similarly, grade 2 IDH–mutant diffuse gliomas showed delayed tumor progression when the drug vorasidenib—an inhibitor of mutant IDH 1 and 2—is prescribed. Mutant IDH inhibition therapy can effectively delay the progression or the time of next intervention for patients. In a phase III INDIGO trial, Mellinghoff et al. reported that vorasidenib significantly improved PFS in grade 2 IDH–mutant glioma patients, demonstrating functional genotype-directed therapy [14]. To compare these findings, Ntafoulis et al. reported that ex vivo drug sensitivity screening using patient-derived glioma cultures predicted the temozolomide response more specifically than the MGMT status, displaying how functional tumor testing may refine stratification where biomarkers cannot accurately indicate treatment paths [15]. Further, in pediatric patients with BRAF V600–mutant low-grade gliomas, Bouffet et al. reported an improved PFS by using a targeted drug combination of dabrafenib and trametinib to slow cell mutation in cases with MAPK pathway activation [16]. Sagerer et al. reviewed focal adhesion kinase (FAK) targeting across CNS tumors and noted that although FAK has been investigated due to its overexpression, clinical glioblastoma trials have failed to demonstrate the benefit in its suppression [17]. These examples display the shift toward genome-directed therapies and how identified epigenetic markers can promote tumor-specific treatments (Table 1). The modern classification of diffuse gliomas is a template for the impact that mature neuro-oncology has, as diagnosis and grading for such tumors are now organized around core molecular features rather than morphology alone.

**Table 1:** Summary of mentioned developments toward biomarker-guided stratification of gliomas that influenced tumor classification, prognosis, and treatment [6,13,14,15,16,17].

Source/Year	Finding	Biomarker	Therapy	Impact
Hegi et al. (2005) [13]	Glioblastoma patients with MGMT promoter methylation benefit more from temozolomide.	MGMT promoter methylation	Temozolomide	Established an epigenetic stratification for glioblastoma multiforme.
Louis et al. (2021 WHO CNS5) [6]	WHO 2021 elevates molecular diagnostics in CNS tumor classification.	IDH status, 1p/19q codeletion		Makes glioma diagnosis more biologically defined compared to morphology.
Bouffet et al. (2023) [16]	Targeted combination drug therapy improves outcomes in pediatric BRAF V600–mutant gliomas.	BRAF V600 mutation	Dabrafenib + trametinib	MAPK/genotype-directed therapy changing management in pediatric gliomas.
Mellinghoff et al. (2023) [14]	Mutant IDH inhibition delays progression in grade 2 IDH–mutant gliomas.	IDH1/2 mutation	Vorasidenib	Landmark genotype therapy demonstrates improved PFS and delayed time to next intervention.
Ntafoulis et al. (2023) [15]	Ex vivo drug sensitivity screening can predict temozolomide responses more specifically than MGMT.	Functional ex vivo TMZ sensitivity	Temozolomide	Functional testing can now refine stratification when single biomarkers are not sufficient/present in the patient.
Sagerer et al. (2025) [17]	Despite overexpression, FAK-targeting has not consistently produced benefits in GBM trials.	FAK marker	FAK inhibitors	Displays that the presence of a biomarker does not indicate therapeutic dependence, emphasizing biological selection.

Meningiomas:

Meningiomas remain predominantly surgical tumors, with radiotherapy used selectively for residual, recurrent, or higher-grade disease. Biomarker-informed and emerging therapies are currently most relevant in refractory or biologically higher-risk cases rather than as replacements for standard local treatment (Table 2). Meningiomas are the most molecularly mature among benign brain tumors, with studies confirming that methylation groupings outperform the WHO grading. Sahm et al. performed a multicenter retrospective analysis which later showed that DNA methylation-based meningioma classes predicted both tumor recurrence and prognosis more effectively than the WHO grade [10]. This is supported by the work by Nassiri et al., who developed a methylation-based nomogram to predict early recurrence risk using molecular variables [11]. In 2025, Landry et al. provided a translational step using a multicenter analysis that reported a next-generation epigenetic-based recurrence which outperformed the WHO 2021 grade for early postoperative recurrence prediction. This framework integrated methylation-based post-surgical recurrence and radiotherapy (RT) sensitivity into four categories (low/high recurrence risk × RT sensitive/resistant) in a cohort of about 2000 meningioma patients [18].

A study by Clark et al. identified frequent non-NF2 driver alterations—including *TRAF7*, *AKT1 (E17K)*, *KLF4 (K409Q)*, and *SMO*—in grade 1 meningiomas, expanding on the previous NF2/22q loss that defined said tumor type [19]. WHO summaries further emphasized that Telomerase Reverse Transcriptase (TERT) promoter mutation and CDKN2A/B homozygous deletion are independent criteria for WHO grade 3 meningioma designation, regardless of tumor morphology [12]. Hirano et al. then provided a risk stratification study which focused on three defined molecular groups in posterior fossa meningiomas: Group A (no Merlin pathway alterations), Group B (NF2 mutation/22q loss without high-risk copy-number alterations), and Group C (high-risk copy-number alterations). Group C (comprising 16/132 (12%) of population) showed significantly worse PFS, even in cases where gross total resection was done; as a result, Hirano thereby demonstrated that the extent of surgical resection cannot entirely offset risks from copy-number alterations [20].

In clinical practice, immunohistochemistry also contributes to diagnostic and prognostic inferences, as specific protein identifiers in the tumor further specify the treatment that would have a higher impact. Severi et al. reported that a practical marker in meningioma was receptor targeting via somatostatin receptor 2 (SSTR2) expression, in that peptide receptor radionuclide therapy (PRRT) was beneficial for tumor control in advanced meningiomas overexpressing SSTR2 [21]. Norden et al. evaluated pasireotide LAR (a multireceptor somatostatin analog evaluated for antiproliferative activity in somatostatin receptor 3 (SSTR3)) in recurrent meningioma using PFS-6 endpoints as a measure of efficacy. They observed a specific marker case in which SSTR3 expression predicted favorable PFS and OS in collective analyses, compared to no observable results from octreotide uptake (another somatostatin analog that instead targets SSTR2 and SSTR5) [22]. Further, Graillon et al. reported a phase II CEVOREM trial in which a drug mixture of everolimus and octreotide was used in meningiomas with somatostatin receptor expression and PI3K/Akt/mTOR (a cell growth/signaling) pathway activation. They observed a PFS-6 of 55%, with the median growth rate decreasing after treatment [23].

Another principal marker therapy development is in NF2 mutant meningiomas, as identified by Brastianos et al. They conducted a phase II trial of focal adhesion kinase (FAK) inhibition (using the GSK2256098 inhibitor) in meningiomas with somatic NF2 mutations and reported that the study demonstrated the goal pathway of molecular eligibility and mechanism, resulting in an interpretable efficacy endpoint [24]. Bi et al. reported a phase II trial which used repeated treatments of nivolumab (anti-PD-1) in recurrent grade 2 and 3 tumors. They reported a PFS-6 of 42.4% and a median OS of 30.9 months; however, they did not have valid comparators for their data, and selected tumors had extremely low mutation burden [25]. In a separate phase II study, Brastianos et al. evaluated pembrolizumab in recurrent high-grade meningiomas with elevated PD-L1 expression and found a PFS-6 (PFS at 6 months post-resection) value of 0.48 and a median PFS of 7.6 months [26].

Kaley et al. evaluated a meningioma treatment that used the drug sunitinib in progressive atypical meningiomas to target decreased VEGFR2 expression. They reported a PFS-6 of 42% and a median PFS of 1.4 months in VEGFR2-negative expression, compared to a PFS of 6.4 months in tumors with positive VEGFR2 expression, illustrating that immunohistochemistry markers may stratify treatment outcomes [27]. Retrospective evidence also suggests anti-angiogenic activity for bevacizumab in recurrent/progressive meningioma. Lou et al. reported a PFS-6 of 86% in a heavily pretreated cohort, supporting bevacizumab as a disease-stabilizing option in selected refractory cases [28]. Raizer et al. then conducted a study which evaluated PTK787/ZK 222584 (vatalanib—a PDGF/VEGF pathway target) and reported stable disease response in 68.2% of patients, with a grade 2 PFS-6 of 64.3% and a grade 3 PFS-6 of 37.5%; however, several proposed marker-based meningioma treatments have not been as successful in establishing clinically applicable outcomes [29]. Wen et al. tested imatinib (a PDGFR inhibitor) in a phase II study on recurrent meningiomas and found that there were no objective responses and 6-month PFS was limited, illustrating that the target PDGFR coexpression did not translate into a significant clinical benefit [30]. Similarly, Norden et al. tested epidermal growth factor receptor (EGFR) inhibitors (gefitinib and erlotinib) in recurrent meningioma and found no objective imaging responses [31]. Further, Reardon et al. evaluated the drug combination of imatinib and hydroxyurea, which had the most successful radiographic response on stable disease though the outcome varied highly on tumor grades, underscoring the need for integrated trial variable stratification when interpreting diseases control [32]. Ji et al. conducted a study which reported on a phase III trial of mifepristone (S9005) in response to the presence of a progesterone receptor compared with a placebo in unresectable meningioma and found no significance to PFS or OS [33]. Song et al. did a further study which reported that SLC7A1 was highly expressed in high-grade meningioma tumors and was associated with malignant phenotypes. Proliferation and invasion by the tumor were reduced via functional knockdown using SLC7A1-FOXM1/E2F4 axis, with the molecule AZ628 showing anti-tumor effects both in vitro and ex vivo models, as well as in organoid models [34].

**Table 2:** Summary of referenced biomarker-informed therapeutic selection and novel risk stratifications in meningiomas [10,11,12,18,19,20,21,22,23,24,25,26,27,28,29,30,31,32,33,34].

Source/Year	Finding	Biomarker	Therapy	Impact
Wen et al. (2009) [30]	PDGFR-targeting phase II imatinib in recurrent meningioma showed no objective responses.	PDGFR/PDGFR target expression	Imatinib	Receptor or target presence alone may not result in clinical benefit.
Nordon et al. (2010) [31]	Phase II EGFR inhibitors (gefitinib and erlotinib) in recurrent meningioma had no objective imaging responses.	EGFR pathway targeting	Gefitinib and Erlotinib	Receptor inhibition without a predictive biomarker was rarely transformative in meningioma.
Reardon et al. (2012) [32]	Phase II imatinib and hydroxyurea reported disease control; outcomes varied by grade.	Tumor grade as a stratifier	Imatinib and Hydroxyurea	Displayed the need for integrated stratification when interpreting tumor endpoints.
Lou et al (2012) [28]	Retrospective bevacizumab series in recurrent meningioma showed PFS-6 of 86% in a heavily pretreated cohort.	Angiogenic/VEGF pathway rationale	Bevacizumab	Supports anti-angiogenic disease stabilization, but without a validated predictive biomarker
Clark et al. (2013) [19]	Identified frequent non-NF2 driver alterations (TRAF7, AKT1 E17K, KLF4).	TRAF7; AKT1 (E17K); KLF4 (K409Q); SMO; NF2/22q	-	Defined molecular subtypes and provided target pathways and biologic stratification.
Kaley et al. (2014) [27]	Phase II sunitinib in progressive atypical/anaplastic meningioma; VEGFR2 expression related to PFS.	VEGFR2 expression (IHC stratifier)	Sunitinib	Met primary endpoint (PFS-6 42%); VEGFR2 IHC median PFS (1.4 months VEGFR2-negative vs. 6.4 months VEGFR2-positive), supporting marker interpretation.
Raizer et al. (2014) [29]	Phase II PTK787/ZK 222584 (vatalanib) targeting PDGF/VEGF pathway; reported high rates of stable disease with grade-stratified outcomes.	PDGF/VEGF pathway target rationale and WHO grade stratification	Vatalanib (PTK787/ZK 222584)	Supported pathway-targeting feasibility but emphasized grade-dependent efficacy.
Norden et al. (2015) [22]	Pasireotide LAR in progressive tumor limited efficacy, with analyses linking SSTR3 to more favorable outcomes.	SSTR3 expression	Pasireotide LAR	Receptor-subtype correlation (SSTR3) may matter more than the somatostatin pathway.
Ji et al. (2015) [33]	Phase III mifepristone vs. placebo in unresectable meningioma showed no improvements.	Progesterone receptor	Mifepristone	Hormone-receptor model did not result in a clinical benefit.
Sahm et al. (2017) [10]	Multicenter analysis showed DNA methylation classes predicted recurrence more accurately than WHO grade.	DNA methylation class	-	Established methylation grouping as a prognostic framework.
Nassiri et al. (2019) [11]	Methylation-based recurrence model and nomogram integrating molecular and clinical factors.	DNA methylation predictor	-	Moved methylation prognostics toward individualized recurrence risk prediction.
Graillon et al. (2020) [23]	Phase II trial using everolimus and octreotide in meningioma with receptor expression and pathway activation.	Somatostatin receptor; PI3K/Akt/mTOR pathway activation	Everolimus and Octreotide	Biomarker-based trial, combining receptor and signaling-pathway context.
Jamshidi et al. (2021-WHO CNS5) [12]	Introduced molecular criteria that qualified meningioma as WHO grade 3 regardless of morphology.	TERT promoter mutation; CDKN2A/B deletion	-	Formalized molecular upstaging where biology can override histology.
Bi et al. (2022) [25]	Phase II nivolumab in recurrent grade 2 or 3 meningioma. Reported limited interpretability and noted low TMB.	Immune context markers	Nivolumab (anti-PD-1)	Immune therapy signals may be constrained by tumor biology and trial comparators.
Brastianos et al. (2022) [26]	Phase II pembrolizumab in recurrent meningioma with PD-L1 expression.	PD-L1 expression context	Pembrolizumab (anti-PD-1)	Some high-grade meningiomas may benefit from immune therapy.
Brastianos et al. (2023) [24]	Phase II FAK inhibitor in NF2-mutated meningiomas demonstrated biomarker-defined eligibility.	Somatic NF2 mutation	GSK2256098 (FAK inhibitor)	Molecularly selected therapy based on pathway dependency.
Severi et al. (2024) [21]	PRRT showed disease control signals in advanced refractory meningiomas overexpressing SSTR2.	SSTR2 overexpression/positive SSTR imaging	Peptide receptor radionuclide therapy (PRRT)	Defined a practical biomarker axis (SSTR2) enabling receptor-targeted radionuclide treatment.
Landry et al. (2025) [18]	Validated an epigenetic-based recurrence predictor, enabling RT sensitivity risk grouping.	Next-generation DNA methylation recurrence predictor	Support for RT selection	Advanced methylation from prognostic to clinically interpretable categories.
Hirano et al. (2025) [20]	Posterior fossa meningiomas stratified into molecular groups; high-risk CNA group had worse PFS after GTR.	Merlin pathway/NF2–22q status with high-risk CNAs	-	Surgical extent of resection cannot fully offset biologic risks driven by CNAs.
Song et al. (2025) [34]	SLC7A1 was expressed in high-grade tumors; knockdown reduced malignant phenotypes and linked regulatory axis.	SLC7A1 and downstream FOXM1/E2F4 axis	AZ628	Provided a candidate molecular dependency and therapeutic axis supported by functional testing.

Schwannomas:

Schwannomas are also predominantly managed with local therapy—especially surgery and radiation—therefore, emerging molecular therapies are most relevant when treatment morbidity is high, when residual or progressive disease persists, or in NF2-related disease where repeated tumor development limits purely local management. Schwannomas, particularly vestibular schwannomas (VS), are benign tumors where morbidity is often defined by functional deficits and treatment trade-offs. Molecular therapies for schwannomas are increasingly being used to either stratify tumor biology or identify tumor dependencies, including pathways that can be combined with local therapies (Table 3) [35]. A study by Zhao et al. developed mouse models for VS to enable hearing testing and reported that mesenchymal–epithelial transition factor (cMET) blockade with crizotinib (tyrosine kinase inhibitor) enhanced tumor radiosensitivity. They increased tumor DNA damage to levels similar to those achieved through high-dosage radiation without adverse hearing effects. They also reported an elevated hepatocyte growth factor (HGF) and cMET activation in human tumors, showing an inhibition of tumor growth in ex vivo brain slice cultures and establishing a process from pathway activation to targeted radiosensitizers and patient function preservation. Based on these findings, Zhao et al. strengthened their translational relevance in human patient application by evaluating elevated HGF expression and cMET activation in NF2-associated VS. This study showed that cMET blockade inhibited the growth of schwannomas in organoid brain cell cultures, providing support that the activation of the HGF/cMET axis supported cMET inhibition as an adjunct to RT to preserve patients’ functional ability [36]. The most established biomarker-based treatment in VS is in NF2-related schwannomatosis, where the studied tumors often show angiogenic signaling, as reported by Plotkin et al. where they evaluated targeted vascular endothelial growth factor (VEGF). VEGF blockade via bevacizumab improved patient hearing and decreased tumor volume, consistent with an anti-angiogenic mechanism that can be translated into a functional benefit [35]. More recent prospective data also support bevacizumab as a function-preserving therapy in NF2-related vestibular schwannoma. In a multicenter phase II maintence study, Plotkin et al. reported high rates of hearing and tumor stability over 18 months, supporting hearing preservation as a key therapeutic endpoint alongside volumetric control [37].

A study conducted by Landry et al. performed a multi-omic study (using the combination of DNA methylation and RNA sequencing) to analyze VS. They identified two molecular subgroups, immunogenic and proliferative tumors, each distinguished by their immune microenvironment and differential target expression (including PD-1/CTLA-4 immune networks, MEK pathway signaling, and epithelial–mesenchymal transition (EMT)). This has since nominated subgroup-aligned therapeutic strategies including an immune checkpoint blockade and MEK-pathway inhibition for further trials on schwannomas [38]. In a 2025 study, Gregory et al. compared the tumor-immune microenvironments of VS versus meningiomas in NF2-related schwannomatosis and found that schwannomas were more immune-cell rich yet exhibited a less activated immune state (indicating relative functional inactivity). This provided supportive evidence that immune activation regulation may be beneficial in schwannoma treatment, displaying the need for the betterment of immunomodulatory therapies for NF2-related tumor management [39].

In 2012, a phase II trial by Karajannis et al. on NF2 patients with progressive VS used 3D volumetric MRI and audiograms to test lapatinib (a dual EGFR/ErbB2 tyrosine kinase inhibitor). From their results, they observed volumetric responses in 4/17 patients and hearing responses in 4/13 with minor overall toxicity. This provided preliminary evidence to support ErbB-family signaling dependency as a therapeutic approach in schwannoma management and functional preservation; however, their results were limited by a small sample size [40]. Two years later, Karajannis et al. published an article on their prospective phase II study which tested everolimus (an oral mTORC1 inhibitor) in NF2 patients with progressive VS based on the biomarker rationale that merlin deficiency activates mechanistic target of rapamycin (mTOR) signaling. They observed no volumetric or hearing responses, and the trial was therefore stopped early in accordance with their ethical guidelines. They thereby concluded that mTORC1 inhibition via everolimus was ineffective for progressive NF2-associated VS treatment [41].

**Table 3:** Summary of mentioned schwannoma biomarker-guided therapies, including key studies linking molecular stratification/pathway activation to functional outcomes [35,36,37,38,39,40,41].

Source/Year	Finding	Biomarker	Therapy	Impact
Plotkin et al. (2009) [35]	In NF2 tumors with progressive VS, VEGF blockade was associated with hearing improvement and reduced tumors, indicating that systemic therapy can change functional outcomes.	Angiogenic signaling and VEGF pathway dependency	Bevacizumab (anti-VEGF)	Established proof for the principle that a biologic axis (angiogenesis) aligned with a tumor volumetric decrease and hearing response.
Karajannis et al. (2012) [40]	Reported volumetric responses and hearing responses in a subset of NF2 patients on treatment.	ErbB signaling (EGFR/ErbB2 axis)	Lapatinib (dual EGFR/ErbB2TKI)	Supported a marker-based benefit beyond VEGF, that pathway inhibition can yield hearing and volumetric benefits.
Karajannis et al. (2014) [41]	Prospective phase II trial in NF2 VS testing mTOR-pathway; observed no volumetric or hearing responses, and the study met early stopping criteria.	NF2/merlin loss and PI3K/AKT/mTOR signaling	Everolimus (mTORC1 inhibitor)	Not all pathway targets can be translated; mTORC1 inhibition was not effective for progressive NF2-VS.
Zhao et al. (2018) [36]	Developed a cerebellopontine angle (CPA) schwannoma mouse model showed cMET blockade (crizotinib) increased DNA damage, demonstrated elevated HGF expression and cMET activation in human NF2-associated VS, and showed cMET blockade inhibited growth in ex vivo cultures.	HGF/cMET axis activation (elevated HGF; activated cMET)	Crizotinib (cMET blockade) and RT. Patient-derived ex vivo	Identified a mechanistic radiosensitizer strategy: pathway activation to targeted adjunct for dose reduction and functional preservation; strengthened translational chain from human markers to target inhibition and growth control, supporting cMET inhibition.
Plotkin et al. (2023) [37]	Multicenter phase II maintenance bevacizumab study showed high rates of hearing and tumor stability over 18 months in NF2-related VS	Functional endpoing framework (hearing preservation, volumetric control)	Bevacizumab	Supports hearing preservation as a major treatment endpoint alongside tumor stability in NF2-related VS
Landry et al. (2023) [38]	Multi-omic profiling (DNA methylation + RNA) identified two VS subgroups (immunogenic vs. proliferative) with distinct microenvironment composition and subgroup therapeutic directions.	Methylation and RNA subgroups	Immune checkpoint blockade; subgroup MEK-pathway inhibition	Shifted therapy toward biology-stratified trials, using molecular subgrouping to match interventions.
Gregory et al. (2025) [39]	In NF2-related schwannomatosis, VS showed immune-cell enriched microenvironments, suggesting that having immune infiltration may not imply effective anti-tumor immunity.	Immune enrichment with suppression	Immunomodulatory and reprogramming	Supported immunotherapy logic that prioritizes immune activation for NF2-related VS.

Neurofibromatosis (NF)-Related Tumors:

NF-related tumors can provide one of the clearest settings in which individualized therapies may be clinically necessary, as patients often develop multiple tumors and repeated local treatments alone may become insufficient. Neurofibromatosis syndromes are genetic tumor predisposition conditions where patients develop peripheral nerve sheath and intracranial tumors. In such tumors, both the tumor type and genome-defined biology must be used in determining diagnostic criteria, surveillance, and trial eligibility (Table 4). An international consensus update led by Plotkin et al. reinvestigated diagnostic criteria for NF2 and schwannomatosis, incorporating both genetics and gene-based naming to better establish management pathways [42]. Early prospective evidence for MEK inhibition in NF1-associated plexiform neurofibromas was provided by Dombi et al, who reported confirmed partial responses in 17 of 24 children (71%) together with clinical improvement in pain, disfigurement, and functional impairment, establishing selumetinib as a clinically meaningful systemic therapy [43]. Longer-term data from Gross et al. further supported sustained symptom benefit from ongoing selumetnib therapy (including pain management), emphasizing that long-term benefits required constant monitoring of efficacy and chronic tolerability [44]. Additionally, the FDA approved mirdametinib (a MEK inhibitor) as of 11 February 2025 for adults and children >2 years of age; the drug targets plexiform neurofibromas (PN) in people with neurofibromatosis type 1 (NF1) when tumors are symptomatic and cannot be completely resected with surgery [45]. Additional support for MEK-pathway inhibition comes from a phase II trial of mirdametinib in adolescents and adults with NF1-related plexiform neurofibromas. Weiss et al. reported a 42% partial response rate together with durable reductions in pain, supporting mirdametinib as another clinically relevant systemic option in this setting [46]. In a study by Fisher et al., cabozantinib was tested in NF1-associated plexiform neurofibromas, producing a tumor volume reduction and improvement in patient pain. This study thereby added to the currently available microenvironment modulation options for MEK-intolerant patients [47]. Mechanistically, NF2/merlin loss continues to highlight Hippo-pathway vulnerability as a therapeutic direction. Recent preclinical work in NF2-deficient schwannoma showed that TEAD-pathway inhibition reduced cell growth and that combined PAK and TEAD inhibition produced synergistic effects, supporting pathway-based targeting in NF2-related tumors [48].

A study by Jordan et al. reported a prospective phase II trial of vistusertib (a dual mTORC1/2 inhibitor) in patients with NF2 and progressive meningiomas. The study design was based on constructive mTOR pathway activation in NF2-deficint tumors. They observed a partial response in 1/18, stable disease in 17/18 patients, and treatment-related adverse effects occurred in about 78% of participants, causing half of the participants to discontinue therapy due to the side effects [49]. Chang et al. performed a drug evaluation in NF2-defficient schwannoma and meningioma models, reporting that brigatinib has potent anti-tumor activity via the inhibitions of multiple receptor tyrosine kinase (RTKs) and other kinases through non-ALK methods. From their experiment, they reported tumor regression and shrinkage in in vivo meningioma modeling and noted an initiation of a clinical trial to further assess brigatnib in NF2-associated tumors [50]. Gregory et al. compared tumor-immune microenvironments of meningiomas and vestibular schwannomas using publicly available transcriptomic datasets to identify shared drug targets relevant to NF2-related schwannomatosis. They reported that immune cells comprised a large portion of VS microenvironment compared to meningiomas, yet multiple immune subtypes were more active in meningiomas, consistent with an enriched schwannoma immune context. They also identified kinase targets across multiple cell types in both tumors and proposed that several drugs (including bosutinib, sorafenib, mitoxantrone, and nintedanib) should be explored, while emphasizing that the tumor type may influence clinical translation [39]. Lastly, a study conducted by Wegscheid et al. reviewed human-induced pluripotent stem cells modeling for NF1 and argued that such cells which were patient-derived offered opportunities to study why individuals with the same germline NF1 alteration developed heterogeneous neurological phenotypes. This functioned as NF1 functional precision approach, which was also applicable to further NF2 trials for PitNETs [51].

**Table 4:** Summary of referenced key contributors and landmark studies that contributed to current personalized biomarker therapies in NF-related tumors [39,42,43,44,45,46,47,48,49,50,51].

Source/Year	Finding	Biomarker	Therapy	Impact
Dombi et al (2016) [43]	Early selumetinib trial in NF1-related plexiform neurofibromas showed confirmed partial responses in 71% and clinical improvement in morbidity-related outcomes	MEK pathway	Selumetinib	Established MEK inhibition as a clinically meaningful systemic therapy in NF1-PN.
Wegscheid et al. (2018) [51]	Reviewed NF1 human stem cell/iPSC modeling; argued that patient-derived iPSCs can explain why identical genotypic NF1 variants yield heterogeneous phenotypes.	Functional precision modeling platform	Platform (no single therapy)	Positioned iPSCs as an NF1 functional approach to support mechanistic stratification and preclinical screening.
Gross et al. (2020) [44]	Phase II trial; most children with NF1 neurofibromas had tumor shrinkage and clinical benefit on selumetinib.	MEK pathway	Selumetinib (MEK inhibitor)	Established MEK inhibition as clinically meaningful systemic therapy in NF1-PN.
Fisher et al. (2021) [47]	Cabozantinib showed activity in NF1-related neurofibromas with tumor volume and pain reduction.	Multi-kinase signaling	Cabozantinib	Demonstrated clinically meaningful activity beyond MEK.
Chang et al. (2021) [50]	Systematic drug evaluation in NF2-deficient meningioma and schwannoma models; brigatinib had potent anti-tumor activity via inhibition of multiple RTKs.	Multi-RTK/kinase dependencies in NF2-deficient tumors	Brigatinib	Preclinical proof supporting translation to an NF2-tumor clinical trial (NCT04374305).
Weiss et al (2021) [46]	Phase II mirdametinib trial in adolescents and adults with NF1-related plexiform neurofibromas showed 42% partial response	MEK pathway	Mirdametinib	Supports mirdametinib as a systemic option in NF1-PN, especially with high symptom burden
Plotkin et al. (2022) [42]	International consensus updated diagnostic criteria for NF2 and schwannomatosis, incorporating genetics and recommending gene-based naming.	Genetics-first diagnosis	Management framework	Molecular criteria can justify diagnosis, surveillance, and trial eligibility rather than phenotype alone.
Jordan et al. (2023) [49]	Phase II trial in NF2 with progressive meningiomas; most showed no change with limited tolerability (78%) and ~50% discontinued; did not meet prespecified primary endpoint.	mTOR activation in NF2-deficient tumors	Vistusertib (mTORC1/2 inhibitor)	Signals of disease control and translation constrained by toxicity—trial endpoint not met.
Benton et al (2024) [48]	TEAD inhibition reduced growth and synergized with PAK inhibition	Hippo/TEAD pathway vulnerability in NF2 deficiency	TEAD-pathway inhibition and PAK inhibition	Supports Hippo-pathway targeting as a therapeutic direction in NF2-related tumors
FDA (11 February 2025) [45]	U.S. FDA approved mirdametinib for adults and children ≥2 with symptomatic NF1 plexiform neurofibromas not possible to completely resect.	MEK pathway	Mirdametinib	Regulatory access for MEK inhibition across adult + pediatric NF1-PN.
Gregory et al. (2025) [39]	Compared meningioma vs. VS time. VS had higher immune cells, but subtypes predicted more functionally active in meningioma and identified kinase targets	Immune microenvironment state and kinase targeting	Repurposing candidates (bosutinib, sorafenib, mitoxantrone, nintedanib)	Defined enriched but suppressed VS immune contexts, highlighting why targets may not translate uniformly across NF2 tumor types.

Pituitary Neuroendocrine (PitNET) Tumors:

Such tumors are increasingly treated as lineage-defined neuroendocrine tumors, where immunohistochemical markers are part of tumor classification and therefore inform prognosis and treatment planning (Table 5). A study by Asa et al. summarized the WHO 2022 PitNET framework, emphasizing the use of transcription factor histochemistry (including factors PIT1, TPIT, and SF1) to define tumor lineages and subtypes with distinct morphological and molecular differences, which acted as a stratifier for PitNET families [52]. Ma et al. reported that for corticotroph tumors, recurrent USP8 mutations represented a molecular driver class, for which frequent variants in ACTH-secreting PitNETs linked said alterations to EGFR signaling dysregulation and ACTH overproduction [53]. For aggressive pituitary tumors treated with temozolomide, MGMT assessment has been widely studied as a predictive tumor marker, though this method is still not ideal. A study by Kontogeorgos et al. evaluated MGMT as a prediction method for aggressive PitNETs, reflecting the appeal and limitations of MGMT-guided temozolomide selection [54]. Yu et al. identified that one of the most relevant advances in organoid-based testing in PitNETs was the use of endocrine evaluation. They reported that ceritinib suppressed both growth and ACTH production in PitNET organoids through Akt1 knockdown in the PI3K–Akt pathway, displaying how ex vivo models can integrate functional endpoints alongside tumor control, particularly where actionable driver mutations are not universal [55].

**Table 5:** Summary of discussed molecular and functional precision strategies in PitNETs, including lineage-defining biomarkers, driver alterations, predictive markers, and associated treatments [52,53,54,55].

Source/Year	Finding	Biomarker	Therapy	Impact
Ma et al. (2015) [53]	Reported recurrent USP8 mutations in ACTH-secreting PitNETs, linking them to EGFR signaling dysregulation and ACTH overproduction.	USP8 mutations with downstream EGFR pathway activation.	EGFR-axis targeting proposed for USP8-mutant tumors	Molecular driver class for corticotrope tumors and targeted axis to hormone hypersecretion.
Kontogeogos et al. (2019) [54]	MGMT as a practical predictive marker for temozolomide response, emphasizing standardization limits and the need for alternative markers.	MGMT	Temozolomide (marker-guided selection)	MGMT-guided TMZ use highlighted limitations that constrained clinical translation.
Asa et al. (2022) [52]	Summarized the WHO 2022 PitNET framework; treated as neuroendocrine tumors with IHC-based classification.	Transcription factor IHC (e.g., PIT1, TPIT, SF1)	Lineage-based stratification	Positioned lineage as the primary stratifier for prognostication and therapeutic planning in PitNETs.
Yu et al. (2025) [55]	Developed ACTH-secreting organoids, screened TKIs, and identified ceritinib; mechanistically implicated PI3K–Akt with AKT1 mediator, linking ACTH regulation to Nur77/POMC control.	ACTH output; pathway mediator AKT1 within PI3K–Akt signaling; Nur77/POMC axis	Ceritinib (identified via organoid screening; evaluated for growth and ACTH suppression)	Drug selection aligned with morbidity endpoints (hormone output) alongside tumor control, addressing limited preclinical modeling.

## 4. Discussion

By organizing tumor evidence according to explicit marker–clinical decision relationships, future prospective trial design may improve in tumors that have historically been managed primarily according to anatomy, histology, and feasibility of local control. Importantly, meningiomas and schwannomas remain predominantly surgical tumors, with radiotherapy continuing to play a major role in selected residual, recurrent, or higher-risk cases. In this context, emerging biomarker-informed and individualized therapies should currently be understood mainly as adjunctive approaches, particularly for residual or refractory disease, WHO grade 2 or 3 meningiomas, and syndromic conditions such as NF1 and NF2 in which patients may develop multiple tumors over time. Accordingly, the most immediate clinical value of molecular profiling lies in refining prognosis, stratifying recurrence risk, and identifying patients who may benefit from biomarker-defined research studies or selective targeted treatment strategies beyond the standard of care.

The central implication of the provided evidence is that difficult benign brain tumors are best understood as chronic, biologically heterogeneous diseases. Molecular and functional biomarkers are used primarily to refine prognosis beyond histology, stratify recurrence and progression risk, inform surveillance and radiotherapeutic planning, and prioritize enrollment in biomarker-defined trials where standard local therapies alone may be insufficient or where recurrence limits conventional options [5]. The large amount of evidence for personalized treatment of meningiomas displays how early targeted therapy approaches failed to meet the necessary criteria, while newer forms of methylation-based modeling and biomarker trials have the potential to create clinically implementable tumor stratification [6].

Epigenetic-based classifiers and modeling frameworks provide a structure for post-surgical risk prediction and radiotherapy response estimation in meningiomas, with direct implications for clinical assessment and therapy selection. Radiogenomics and radiomics are methods that translate medical images into quantitative prediction data, allowing for options when tumor tissue is scarce or biopsy is impractical. While the current evidence is early, medicine is traveling toward imaging centered on non-invasive methods of collecting molecular state information. Additionally, patient-derived organoids and ex vivo culture systems can allow for phenotype testing that incorporates tumor heterogeneity, immune microenvironments, and functional outputs. The transition from biomarker discovery to clinically implementable personalized therapies depend on the integration of neurosurgery, molecular pathology, advanced imaging, and clinical trial infrastructure development [56]. Ultimately, surgery and radiotherapy remain the principal treatments responsible for improving outcomes in most patients with difficult benign brain tumors. In current practice, molecular and functional biomarkers are most valuable for refining diagnosis, stratifying risk of progression or recurrence, informing surveillance and radiotherapeutic decision-making, and identifying patients who may be appropriate for biomarker-defined clinical trials or research studies. Personalized therapy is therefore best understood, at present, as an adjunctive framework that complements localized treatment by improving biological stratification and supporting selective recruitment to targeted therapeutic approaches [6].

Novel Technologies: Radiogenomics, AI-Based Biomarkers, and Theranostics:

An additional emerging direction in personalized neuro-oncology is the use of non-invasive imaging to predict clinically relevant molecular states [57,58]. In gliomas, radiomics and deep learning studies have shown that routine preoperative MRI can be used to estimate actionable biomarkers, including MGMT promoter methylation and IDH-wild-type/TERT-promoter-mutant molecular status, thereby linking imaging more directly to diagnostic, prognostic, and treatment-stratification decisions [57,59]. A recent modality-adaptive radiomics study demonstrated biologically consistent MRI for MGMT methylation in high-grade gliomas and showed decision-analytic value for clinical risk stratification, supporting the feasibility of MRI-based biomarker estimation in heterogeneous imaging [57]. A separate multiparametric MRI fusion model integrating radiomics with transformer-based deep learning identified IDH-wild-type, TERT-promoter-mutant gliomas with strong external-validation performance, and stratified overall survival risk [58]. These developments are particularly relevant to the broader theranostic paradigm in neuro-oncology, in which molecular imaging, target identification, and treatment selection are increasingly integrated. Within this framework, receptor-targeted radionuclide therapy in meningioma can be viewed not only as a receptor-based treatment strategy, but also as part of a wider theranostic model that connects imaging-defined target expression with personalized therapeutic planning [59]. Although these approaches remain investigative and require further validation before routine implementation, they strengthen the rationale for integrating imaging, molecular profiling, and biologically informed treatment selection in neuro-oncology [57,58,59].

Limitations:

Despite its notable impact on current medicine, marker-based personalized CNS tumor therapy still has several research gaps. Personalized therapies often have practical limitations, including universal access, treatment turnaround timeframes, clinical application, and small patient populations in clinical trials. The translation of genetic markers into treatment remains limited by the evidence quality, standardization barriers, and high costs that long-term clinical trials need [6]. Many biomarkers remain exploratory, with heterogeneous workflows and limited external validation despite genomic plausibility. There is still a prevalent need for prospective biomarker-defined clinical trials in meningioma and NF2-related tumors, as large methylation-based modeling studies explicitly note limitations due to their retrospective design and their potential selection bias toward more aggressive CNS tumor cases [11]. Similarly, immunotherapy in benign intracranial tumors requires a treatment approach heavily driven by biomarkers. Current PD-1 inhibitor trials in meningiomas show that they may work in a subset of patients, however they remain underpowered and their benefit varies greatly across tumor types. Further steps should not be higher doses of immunotherapy, but rather more precise patient immune phenotyping—including functional state, myeloid biology, and antigen presentation [26]. Finally, functional testing requires standardization and prospective validation to evaluate treatment success relative to patient OS and PFS. Organoids and ex vivo treatments appear promising; however, they require consistent culture methods, reproducible readouts, and dependable demonstrations that they can improve patient outcomes [52].

Future Directions:

Future progress in the personalization of difficult benign CNS tumor treatments will shift from depending on universally effective drug therapies toward a constructive treatment infrastructure that bases clinical decisions on patient biomarkers. The current aims of treatment development are to classify molecular markers into further exploring extent of resection aims, radiotherapeutic selections, surveillance intensity, and trial eligibly rather than using blanket prognostic labels [12]. One of the strongest examples of the mentioned sources that uses a methylation-driven management framework was advanced by Landry et al., who framed methylation-profiling as a tool that can predict post-surgical outcomes and response to radiotherapy. They turned this into a scheme that measured interpretable risk and radiosensitivity ratios in a cohort of nearly 2000 surgical cases [18]. While further infrastructure is needed for such systematic approaches to drug evaluations, it is important to allow for personalization in a system that attempts to use homogenous classification.

## 5. Conclusions

This review synthesizes the evidence that difficult benign CNS tumors require a shift from histological- to biomarker-centered treatment. This shift would decrease diagnostic bias, as it bases tumor grade and treatment on testable markers rather than individual doctors’ perception of tumor location and progression. Despite the current progress, implementation is constrained by the need for prospective biomarker-defined trials, standardized workflows, and molecular classifier-driven immunotherapies. Integrated molecular profiling and functional testing should be used to guide clinically implementable, personalized treatments for difficult benign intracranial tumors beyond surgery.

## Data Availability

No new data were created or analyzed in this study.
